# BDNF and proBDNF Serum Protein Levels in Obstructive Sleep Apnea Patients and Their Involvement in Insomnia and Depression Symptoms

**DOI:** 10.3390/jcm11237135

**Published:** 2022-11-30

**Authors:** Agata Gabryelska, Szymon Turkiewicz, Marta Ditmer, Filip Franciszek Karuga, Dominik Strzelecki, Piotr Białasiewicz, Marcin Sochal

**Affiliations:** 1 Department of Sleep Medicine and Metabolic Disorders, Medical University of Lodz, 90-419 Lodz, Poland; 2 Department of Affective and Psychotic Disorders, Medical University of Lodz, 90-419 Lodz, Poland

**Keywords:** BDNF, OSA, sleepiness, neurotrophins, intermittent hypoxia, insomnia, polysomnography, depression

## Abstract

Introduction: Obstructive sleep apnea (OSA) is a disorder that, apart from somatic sequelae, increases the risk of developing psychiatric conditions. Brain-derived neurotrophic factor (BDNF) signaling pathway is involved in the pathophysiology of depression and insomnia. Therefore, the study aimed to investigate differences in concentrations of BDNF and proBDNF in patients with OSA and healthy individuals, to evaluate diurnal changes of these proteins, and to assess the correlations with psychiatric symptoms. Methods: Sixty individuals following polysomnography (PSG) were divided into two groups based on the apnea-hypopnea index (AHI): OSA patients (AHI ≥ 30; *n* = 30) and control group (AHI < 5; *n* = 30). Participants filled out questionnaires: Beck Depression Inventory (BDI), Athens Insomnia Scale (AIS), and Pittsburgh Sleep Quality Index (PSQI). Peripheral blood was collected before and after PSG. Protein concentrations were measured using ELISA. OSA group was divided into subgroups: AIS (−)/AIS (+) (AIS > 5), PSQI (−)/PSQI (+) (PSQI > 5), and BDI (−)/BDI (+) (BDI > 19). Results: No differences in BDNF and proBDNF protein levels were observed between OSA and the control groups. However, BDNF and proBDNF evening protein concentrations were higher in the AIS (+) and PSQI (+) groups (*p* < 0.001 for all). The BDI (+) group was characterized by lower morning levels of both proteins (*p* = 0.047 and *p* = 0.003, respectively). Conclusions: BDNF signaling pathway might be involved in the pathophysiology of depression and insomnia in patients with OSA. BDNF and proBDNF protein levels might be useful in defining OSA phenotypes.

## 1. Introduction

Obstructive sleep apnea (OSA) is a common sleep-related breathing disorder characterized by recurrent pauses in breathing during sleep due to partial or complete obstruction of the upper airways [1]. Numerous pauses in breathing conduce to intermittent hypoxia (IH), which is closely correlated with systemic inflammation and oxidative stress [2]. Those factors might contribute to OSA comorbidities, like hypertension, other cardiovascular diseases, or metabolic disorders, especially type II diabetes mellitus [3,4,5,6,7,8]. Studies have also highlighted the association between conditions with immune-mediated background and OSA, which indicates some immune system dysregulation occurring in the course of OSA in susceptible individuals [9,10,11,12,13].

Recently more studies have been focusing on the psychiatric aspects of this disorder, i.e., increased prevalence of affective diseases (particularly depression), cognitive impairment, or co-existing insomnia. The prevalence of this condition in patients with OSA in some studies reaches up to 84% [14]; it might greatly affect a patient’s prognosis and overall health as well as affect adherence to treatment and its potential effectiveness [15,16,17]. In one study, the risk of all-cause mortality in the OSA group with comorbid insomnia was 47% higher than non-insomnia OSA controls [18]. Regarding presence of depressive symptoms in patients with OSA, Jehan et al. reported that about 20% of patients with OSA might suffer from a major depressive disorder (MDD) [19]. In a large cohort study, MDD with concomitant OSA was associated with a higher prevalence of suicidal behaviors, defined as attempts or suicidal ideation [20].

Brain-derived neurotrophic factor (BDNF), a neurotrophin (NT) produced in the central nervous system, has the ability to cross the blood-brain barrier, thus, has been widely studied in psychiatric disorders [21,22,23,24]. BDNF regulates the development and functioning of several signal pathways, such as Ras/MAPK, PI3K/Akt, and PKC/PLC. Its premature form (proBDNF) has the opposite activity and leads to the activation of pro-apoptotic factors in neural cells [25]. This NT has already been correlated with numerous disturbances characteristic of OSA, such as neurocognitive decline [26], sleep disturbances [27], or depression [28,29].

There is limited data, with contradicting results regarding BDNF levels in patients with OSA [30,31,32,33]. To the best of our knowledge, no studies up to date have evaluated the concentration of this neurotrophin in the context of depressive and insomnia symptoms in patients with OSA. Therefore, the study aimed to investigate differences in concentration of BDNF and proBDNF in patients with OSA and healthy participants, to evaluate their diurnal changes, and to assess their correlations with insomnia and depression symptoms that might contribute to specific OSA phenotypes.

## 2. Materials and Methods

### 2.1. Sample

The study group included 60 participants referred to the Sleep and Respiratory Disorders Centre in Lodz (Poland) with potential OSA diagnosis. Each participant underwent a standard nocturnal polysomnography (PSG) examination. Based on the apnea-hypopnea index (AHI), patients were divided into the OSA group (*n* = 30; AHI ≥ 30) and the healthy control group (*n* = 30; AHI < 5). The exclusion criteria included inflammatory diseases (e.g., connective tissue diseases or inflammatory bowel diseases), chronic respiratory diseases (e.g., bronchial asthma or chronic obstructive pulmonary disease), any infection within one month of blood collection, diagnosis of cancer (in medical history), diagnosed major neurological conditions, diagnosed psychiatric disorders including insomnia and taking medications affecting sleep (e.g., benzodiazepines and melatonin). The study was approved by the Ethics Committee of the Medical University of Lodz (RNN/432/18/KE). All patients provided written informed consent to participate in the study.

### 2.2. Polysomnography

Patients were admitted to the sleep lab at 21:00 h (±0.5 h) and underwent physical examination (measurement of body mass, height, heart rate, and blood pressure). Standard nocturnal polysomnography was performed by recording the following channels: electroencephalography (C4\A1, C3\A2), chin muscles and anterior tibialis electromyography, electrooculography, measurements of oronasal airflow (a thermistor gauge), snoring, body position, respiratory movements of chest and abdomen (piezoelectric gauges), unipolar electrocardiogram and hemoglobin oxygen saturation (SpO_2_) (Alice 6, Phillips-Respironics). Sleep stages were scored according to the criteria based on the 30-s epoch standard [34]. Apnea was attained with over 90% the airflow reduction for at least 10 s. Hypopnea was defined as at least a 30% reduction of airflow for at least 10 s, accompanied by over a 3% decrease in SpO_2_ or arousal. Encephalography arousals were scored according to the American Academy of Sleep Medicine guidelines [34].

### 2.3. Questionnaires

Questionnaires included three research instruments: Pittsburgh Sleep Quality Index (PSQI), Beck Depression Inventory (BDI), and Athens Insomnia Scale (AIS). They were filled in by each participant in the morning after polysomnography.

#### 2.3.1. Pittsburgh Sleep Quality Index (PSQI)

Self-evaluation questionnaire assessing seven different aspects of sleep in adults. It evaluates sleep quality parameters such as difficulties with falling asleep, problems with maintaining continuity of sleep, functioning during the day, and questions regarding the most frequent causes of sleep disorders over the past four weeks. They all make up the outcome, assessed from 0 to 21 points. Results higher than 5 points indicate low sleep quality and differentiate patients into “poor” and “good” sleep [35,36,37]. This principle was used to determine the cutoff point used to divide patients with OSA into two groups: the PSQI (−) group (PSQI ≤ 5) and the PSQI (+) group (PSQI > 5). PSQI proved to have high internal consistency, as indicated by Cronbach’s alpha of 0.83 [38]. A validated PSQI version in Polish was used in the study [39].

#### 2.3.2. Beck Depression Inventory (BDI)

The self–evaluation questionnaire consists of 21 questions, which assesses the intensification of each depression symptom on a 4-grade scale from 0 to 3 points. All answers are added up, giving a maximum score of 63 points. BDI is a quick and simple screening test. The BDI interpretation divides results into four groups: slight depression (0–13), mild depression (14–19), moderate depression (20–28), and severe depression (29–63) [40,41]. The questionnaire doesn’t define a lack of depression [42]. Its validity and reliability were determined by Cronbach’s alpha of 0.913 [43]. We used 19 points result as a cutoff point to determine OSA groups with poor and intensified symptoms of depression: the BDI (−) group (BDI ≤ 19) and BDI (+) group (BDI > 19), respectively. A validated BDI version in Polish was used in the study [44,45].

#### 2.3.3. Athens Insomnia Scale (AIS)

A questionnaire consisting of 8 questions dedicated to insomnia studies. The first five questions are according to the ICD-10 criterion of insomnia diagnosis, including assessing difficulty with sleep induction, awakening, total sleep time, and overall quality of sleep. The last three items evaluate day consequences of insomnia, such as subsequent day well-being, functioning, and daytime sleepiness [46]. Each question is scored from 0 to 3 points, corresponding to “no problem at all” to “very serious problem”, respectively. Summarizing all items assessed to 24 points in total. Saldatos et al. reported that the cutoff score is 5.5 points in European countries [46], and it was used in the study to determine the AIS (−) group (AIS ≤ 5) and AIS (+) group (AIS > 5). A validated AIS version in Polish was used in the study [47].

### 2.4. Blood Collection and Protein Level Assessment

Peripheral blood samples were collected in the evening before and in the morning following PSG examination into collection tubes with a clot activator. Blood samples were centrifuged immediately following the blood draws at 4 °C. The serum was collected and stored at −80 °C. The serum BDNF and proBDNF protein concentrations were assessed by ELISA kit (Human BDNF (Brain-Derived Neurotrophic Factor) ELISA Kit and Human pro-BDNF (pro-Brain-Derived Neurotrophic Factor) ELISA Kit respectively, FineTest, Wuhan, China). The absorbance was measured at λ = 450 nm wavelength by an absorbance reader (BioTek 800 TS, Agilent Technologies, Santa Clara, CA, USA).

### 2.5. Statistical Analysis

Statistical analysis was performed at a significance level of 0.05 using two-tailed tests. The normality of the distribution of variables was tested with the Shapiro-Wilk test. For variables with a normal distribution, the data is presented as the mean with the standard deviation; for variables with a distribution other than normal, the data is presented as the median with the interquartile range (IQR). Chi-square and Chi-square tests with Yate’s correction were used to assess nominal variables in situations where the size of the smallest group was, respectively: above 15 and in the range of 5–15. Comparisons of independent groups were made using the student’s *t*-test (for variables with a normal distribution) and the Mann-Whitney U test (for variables with a different distribution than normal). Dependent groups were compared with the *t*-student test for dependent variables (for variables with a normal distribution) or Wilcoxon (for variables with a different distribution than normal). Correlations between continuous variables were tested with Spearman’s rank correlation test. The analysis was performed using IBM SPSS Statistics version 28 (2021, Armonk, NY, USA).

## 3. Results

Baseline characteristics and comparison between the control group (*n* = 30) and OSA group (*n* = 30), including demographic data, polysomnography parameters, protein concentrations, and questionnaire results, are shown in Table 1.

**Figure 1:**
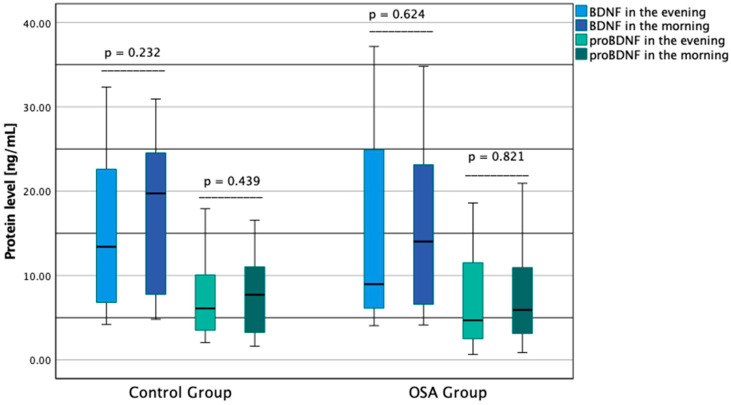
Comparison of diurnal protein levels. BDNF—brain-derived neurotrophic factor; OSA—obstructive sleep apnea proBDNF—premature brain-derived neurotrophic factor.

No differences were found between the morning and the evening protein concentrations in the case of BDNF (*p* = 0.162) and proBDNF (*p* = 0.791) in all participants of the study; similarly, no differences were observed in the control group (*p* = 0.232 and *p* = 0.439 respectively) and the OSA group (*p* = 0.624 and *p* = 0.821 respectively) (Figure 1).

**Figure 2:**
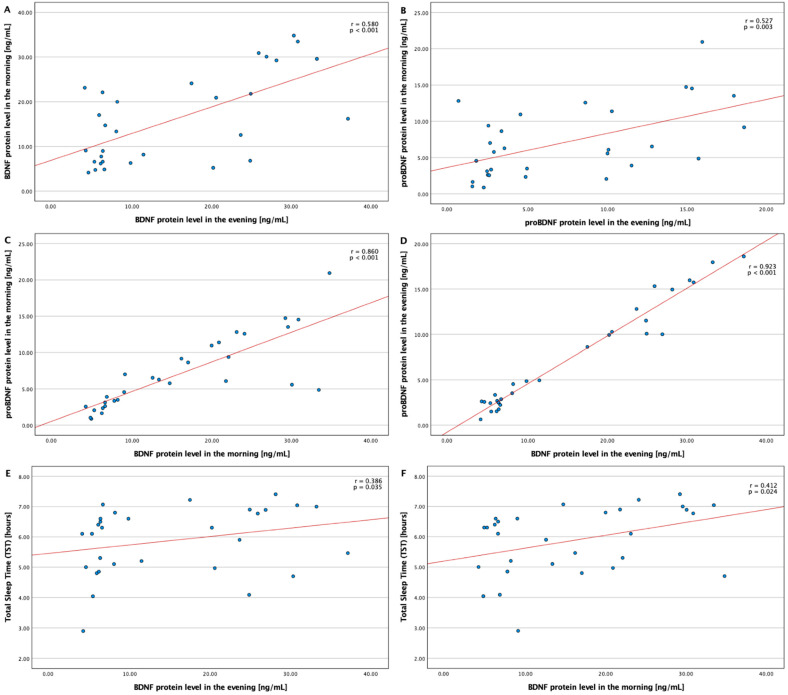
Correlations between BDNF and proBDNF protein levels and chosen parameters BDNF—brain-derived neurotrophic factor; proBDNF—premature brain-derived neurotrophic factor; TST—total sleep time. (**A**)—Correlation between BDNF protein level in the morning and BDNF protein level in the evening. (**B**)—Correlation between proBDNF protein level in the morning and proBDNF protein level in the evening. (**C**)—Correlation between proBDNF protein level in the morning and BDNF protein level in the morning. (**D**)—Correlation between proBDNF protein level in the evening and BDNF protein level in the evening. (**E**)—Correlation between total sleep time and BDNF protein level in the evening. (**F**)—Correlation between total sleep time and BDNF protein level in the morning.

In the OSA group, strong positive correlations between the morning and the evening of BDNF (r = 0.580, *p* < 0.001) and proBDNF (r = 0.527, *p* = 0.003) concentration were observed. Moreover, a very strong positive correlation between BDNF and proBDNF protein concentration in the morning (r = 0.860, *p* < 0.001) and in the evening (r = 0.923, *p* < 0.001), respectively, was achieved. Additionally, BDNF protein concentration positively correlated with total sleep time both in the evening (r = 0.386, *p* = 0.035) and in the morning (r = 0.412, *p* = 0.024) (Figure 2).

**Figure 3:**
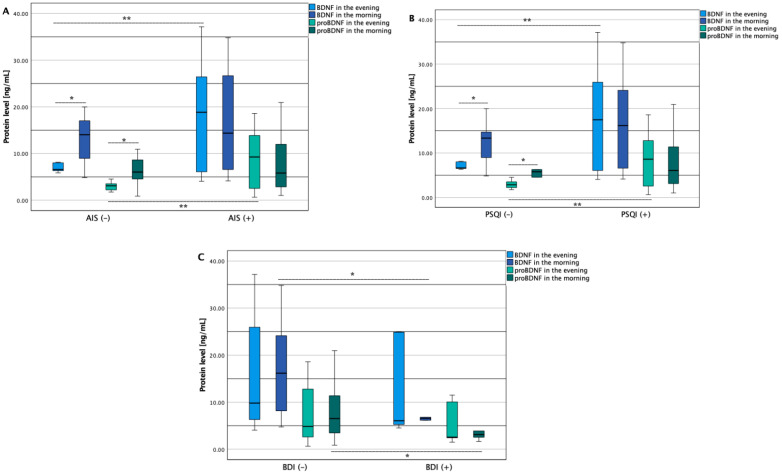
Diurnal and between groups comparisons of BDNF and proBDNF protein levels. AIS—Athens Insomnia Scale; BDI—Beck Depression Inventory; BDNF—brain-derived neurotrophic factor; proBDNF—premature brain-derived neurotrophic factor; PSQI—Pittsburgh Sleep Quality Index; **—*p* < 0.001; *—*p* < 0.05. (**A**)—comparison of BDNF and proBDNF protein levels between participans with low AIS (−) and high AIS (+) scores. (**B**)—comparison of BDNF and proBDNF protein levels between participans with low PSQI (−) and high PSQI (+) scores. (**C**)—comparison of BDNF and proBDNF protein levels between participans with low BDI (−) and high BDI (+) scores.

In further analysis, the OSA group was divided based on results on AIS (≤5 and >5), PSQI (≤5 and >5), and BDI (≤20 and >20), the comparisons between the subgroups and baseline characteristics are shown in Table 2. AIS (+) subjects had increased evening BDNF and proBDNF levels in contrast with its AIS (−) group (18.8 (6.1–26.7) vs. 6.6 (6.2–8.1) *p* < 0.001, and 9.7 (2.5–28.0) vs. 3.1 (2.1–3.8) *p* < 0.001 respectively, Figure 3A). Similar results of evening BDNF and proBDNF were also obtained between PSQI (+) and PSQI (−) groups (17.5 (6.0–26.4) vs. 6.6 (6.4–8.1) *p* < 0.001, and 8.6 (2.5–13.9) vs. 2.9 (2.0–4.0) *p* < 0.001 respectively, Figure 3B). Furthermore, decreased morning BDNF and proBDNF levels were observed in the BDI (+) group compared to the BDI (−) group (6.6 (5.2–14.3) vs. 16.2 (8.0–26.7) *p* = 0.047, and 3.5 (±1.7) vs. 7.8 (±5.1) *p* = 0.003 respectively, Figure 3C).

Furthermore, an increased level of the morning compared to the evening BDNF and proBDNF protein concentrations were achieved in the AIS (−) group (14.0 (8.0–17.8) vs. 6.6 (6.2–8.1) ng/mL *p* = 0.033 and 6.0 (3.6–9.2) vs. 3.1 (2.1–3.8) ng/mL *p* = 0.035 respectively, Figure 3A) and the PSQI (−) group 13.4 (6.9–17.3) vs. 6.6 (6.4–8.1) ng/mL *p* = 0.043 and 5.8 (2.7–8.6) vs. 2.9 (2.0–4.0) ng/mL *p* = 0.046 respectively, Figure 3B), while no differences in protein diurnal levels were observed in the AIS (+), the PSQI (+), the BDI (+) as well as BDI (−) groups (Figure 3).

## 4. Discussion

BDNF signaling pathway might be a contributor to the course of OSA. It seems to influence the risk of developing OSA comorbidities, such as insomnia, cognitive impairment, and depression. In this study, we demonstrated increased evening levels of BDNF and proBDNF in patients with OSA who scored high on questionnaires assessing poor sleep quality and insomnia symptoms (PSQI and AIS, respectively). Moreover, the severity of depression symptoms assessed with BDI was associated with decreased morning serum levels of both proteins in the OSA group. This may suggest the plausible involvement of those proteins in developing mood and sleep disorders in OSA. In addition, serum levels of BDNF and proBDNF are not significantly different between healthy individuals and patients with severe OSA, and no diurnal changes are present.

The majority of evidence indicates that intermittent hypoxia, one of the most damaging effects of OSA, causes decreases in BDNF levels in animal models [48,49,50,51,52,53,54]. Fang et al. studied the influence of CIH (chronic intermittent hypoxia) on neurodegeneration of the optic nerve in mice model, showing decreased BDNF levels. Those changes were reversible after 7,8-dihydroxyflavone (7,8-DHF) administration, an antioxidant [48]. After 7,8-DHF application, oxidative stress was reduced, and BDNF/TrkB/CREB pathway increased in activity, which emphasized the role of reactive oxygen species (ROS) in the impairment of BDNF expression [21,55]. Mice and piglet model of OSA confirmed the involvement of Trk/CREB pathway regulation of the BDNF disruption [51,56]. Another study obtained similar results of BDNF pathway downregulation in the CIH mice model, but it emphasized the role of impaired iron metabolism as a potential pathomechanism [49].

Our study didn’t find any significant differences in BDNF and proBDNF levels between OSA and the healthy control group. Interestingly, most human studies on BDNF in OSA showed no differences in BDNF levels [30,31,32,57,58,59]. On the other hand, Shah et al. showed increased BDNF expression in the soft palate muscles of snorers and patients with OSA [60]. A possible explanation of this paradox is a time of exposure to IH. In studies on animal models time of exposure was between 3 to 12 weeks. OSA is a chronic disease, and it has been affecting patients for many years. This time is sufficient to activate the adaptive mechanisms. It has been suggested that sensorimotor neuropathy may cause upper airway collapse in patients with OSA [61]. Shah et al. showed that neuromuscular injuries caused by vibrating are typical in those groups of patients [62]. The following denervation was correlated with increased BDNF expression in the local environment, the same with swallowing dysfunction. The authors concluded that it might be an adaptation to neuromuscular injuries, which can lead to reinnervation [60]. Flores et al. also observed increased BDNF levels in patients with OSA. They emphasized the role of BDNF in neuroprotection in patients with OSA. In their study, higher BDNF levels correlated with higher oxygen desaturation index and with the Montreal Cognitive Assessment questionnaire score; it suggests that an increase in BDNF concentration might result in an improvement in cognitive functions. Thus, BDNF could be considered as o protective factor against cognitive decline [33]. Arslan et al. also reported the protective role of BDNF in patients with OSA in response to neurodegeneration. In their study, they found increased levels in mild and moderate-to-sever OSA than in healthy participants [63]. Moreover, BDNF levels correlated positively with the hypoxia [63]. In the same study, a similar correlation was also received for hypoxia and neurofilament light chain level (NF-L) [63], which is used as a biomarker of axonal damage in the AD [64]. However, the correlation of BDNF with NF-L in each group was insignificant [63]. The impact of hypoxia-inducible factor 1 (HIF-1) is worth considering in the context of adaptation to IH. We’ve already shown that patients with OSA are characterized by overexpression of HIF-1 [65,66,67], with other groups reporting similar results [68]. HIF-1 is a factor that mediates hypoxia-dependent response. Among the numerous targets, BDNF is one of them [69], and potentially overexpression of HIF-1 may affect the BDNF signaling pathway in patients with OSA, but it needs further research.

One of the main findings of our study is that patients with OSA with poor sleep and insomnia have higher evening BDNF and proBDNF levels. Moreover, the level of both proteins was positively correlated with total sleep time (TST). Even though the effect sizes of these differences in our studies were low, they were in line with Kaminska et al. and More et al., who showed a similar association between daytime sleepiness and BDNF overexpression in OSA [32,70]. In these studies, daytime sleepiness was evaluated by ESS. Yet, the above outcomes contradict the recent results of insomnia studies, where objective sleep and subjective sleep were correlated with lower serum levels of BDNF [71,72,73]. Fan et al. pointed out that decreased BDNF levels characterized insomnia patients with SSD, lower than 6 h compared to insomnia patients with sleep duration ≥ 6 h and controls. Moreover, the SSD group showed impaired neurocognitive functions, which correlated positively with BDNF levels [71]. What is more, Mikoteit et al. investigated subjective insomnia using Insomnia Severity Index (ISI); the severity of symptoms was correlated with decreased BDNF levels. In contrast to Fan’s outcomes, Micoteit et al. showed a correlation between decreased BDNF levels and decreased REM sleep in objective insomnia patients, not with sleep duration [72]. Another study on individuals with insomnia also confirmed the relationship between the severity of subjective sleep impairment and lower serum BDNF levels [73]. Down-regulation of BDNF in insomnia could be explained by hyperactivity of the stress response system and inflammation. Hyperactivity of the hypothalamus-pituitary-adrenal glands axis (HPA) is caused by hyperarousal. In insomnia patients, morning cortisol level is increased [74], and nocturnal melatonin production is diminished [75], which can disturb sleep and its structure, for instance, by REM-sleep changes. Claro et al. emphasized the impact of the stress itself on inflammation and BDNF levels without binding them directly [76]. Thereupon, a positive correlation between proBDNF, BDNF, and total sleep time in OSA and a higher level of expression of studied proteins in OSA individuals with poor sleep quality and insomnia symptoms indicate that patients afflicted with this disease might develop specific compensational mechanisms which are associated with increased expression of studied neurotrophins. Another finding, namely a positive correlation between the level of BDNF and its precursor, which could suggest relatively high activity of the BDNF pathway, might partially corroborate this hypothesis. A possible adaptive mechanism of BDNF pathways upregulation in OSA was described above. This is in line with results form a study where groups did not differ regarding age and sex [33]. In conclusion, BDNF levels may be useful in defining the insomnia phenotype in patients with OSA characterized by excessive daytime sleepiness. It is necessary to understand the complexity of the relationship between sleep disturbances and BDNF. Further research on the exact mechanisms driving this association is warranted.

Circadian clock disruption is a feature property of OSA. Our recent studies confirmed decreased levels of circadian clock proteins, such as period 1 protein (PER1) and aryl hydrocarbon receptor nuclear translocator-like protein 1 (BMAL1), in patients with OSA [77,78]. BDNF protein’s expression and its mRNA show dependence on the circadian rhythm: mRNA and BDNF protein levels are elevated during biological night and day, respectively [79]. In this study, we found higher BDNF and proBDNF levels in the morning than in the evening only in the AIS (−) and the PSQI (−) groups. The lack of significant differences in the protein levels in the AIS (+) and the PSQI (+) groups may indicate a possible connection with impaired circadian rhythm.

OSA is well known for neurobehavioral and cognitive deficits, such as decreased attention and vigilance, phonological problems, irritability, and impairment in executive functions and the long-term memory [26]. Neurocognitive impairment was correlated with decreased BDNF levels in the hippocampus several times in mice models of OSA [49,53,79]. Its pathomechanism is complex; nevertheless, it is based on the IH. Oxidative stress caused by IH damages synapses and neurofilaments directly, including postsynaptic density protein 95 failure, impairs new synaptic connections’ development [49], and inhibits serotonergic signaling. Wall et al. found that mice had impaired hippocampal neuroplasticity after seven days of IH, measured by long-term potentiation (LTP) in the CA1 region but not in the dentate gyrus [80]. Similar outcomes were obtained by Xie et al. [53]. In the latter study’s proposed mechanism, IH and sleep fragmentation directly lead to decreased neuronal excitability, decreased BDNF expression, and enhanced generation of reactive oxygen species [26]. They all impact neurocognitive dysfunction by impairing synaptic plasticity and promoting neuronal apoptosis, which contributes to depression. This could be confirmed to some extent by decreased morning BDNF and proBDNF levels with a medium effect size in the OSA group, with BDI (+) scores observed in our study. It would mean that reduction of BDNF in patients with OSA with depression symptoms may further exacerbate symptoms of this disease and hinder the therapeutic effects of antidepressants.

The main limitation of the study was the small size of the groups. Moreover, insomnia and depression assessments were based on questionnaires, with no other clinical investigation. Additionally, the study design (cross-sectional) prevents a conclusion on the causality. Prospective, interventional trials are necessary to understand the nature of this relationship.

## 5. Conclusions

Results of the study suggest that BDNF and proBDNF may be associated with symptoms of insomnia and depression in patients with OSA. Higher BDNF levels may define OSA phenotypes with comorbid insomnia, provide a better description of this heterogenic disorder, and further support proper treatment decisions. Similarly, lower BDNF and proBDNF levels may define OSA phenotypes with intensified symptoms of depression. However, the lack of differences in those protein levels between OSA and control groups indicates the greater complexity of the relationship. Further research is needed to verify the role of BDNF and its precursor in the development of psychiatric OSA comorbidities and assess the effect of treatment on these proteins.

## Figures and Tables

**Table 1:** Baseline characteristics of the study groups.

		Control Group (*n* = 30)	OSA Group (*n* = 30)	*p*-Value
Demographic data	Sex [M (%)/F(%)]	17 (56.7%)/13(43.3%)	27 (90%)/3(10%)	0.007
	Age [years]	46.5 (36.8–58.5)	59.5 (48.8–67.0)	<0.001
	BMI [kg/m^2^]	27.6 (24.3–30.7)	36.0 (31.2–39.4)	<0.001
Polysomnography	TST (h)	6.0 ± 0.9	5.9 ± 1.1	0.994
	REM (h)	1.2 ± 0.5	1.2 ± 0.6	0.408
	NREM (h)	4.8 ± 0.7	4.7 ± 0.9	0.525
	Sleep Efficiency (%)	85.2 (76.4–91.1)	83.8 (70.3–87.8)	0.412
	Sleep onset latency (min)	19.5 (8.1–31.8)	15.8 (7.4–29.4)	0.647
	Sleep Maintenance Efficiency (%)	93.5 (82.6–97.4)	89.1 (77.2–92.2)	0.205
	REM latency (min)	102.5 (80.1–144.0)	97.3 (56.4–164.3)	0.595
	Arousal index (events/h)	9.4 (6.0–14.2)	24.8 (18.3–34.5)	<0.001
	AHI (events/h)	2.5 (1.3–4.2)	49.0 (37.5–71.3)	<0.001
	AHI in REM sleep (events/h)	4.0 ± 5.2	48.4 ± 25.9	<0.001
	AHI in NREM sleep (events/h)	1.6 (1.1–3.7)	47.6 (36.9–69.0)	<0.001
	Total nuber of desaturations	10.5 (6.3–18.5)	248.0 (203.0–383.5)	<0.001
	Desaturation Index	2.5 ± 1.8	56.6 ± 26.3	<0.001
	Mean SpO2 of desaturations during sleep	91.0 (90.0–92.4)	86.0 (81.9–88.5)	<0.001
Questionnaires	AIS	10.0 (7.0–14.0)	11.0 (7.5–13.5)	0.902
	PSQI	10.0 (7.0–11.0)	11.0 (7.0–13.0)	0.378
	Subjective sleep quality (component 1 PSQI)	2.0 (1.0–2.0)	2.0 (1.0–2.0)	0.482
	Sleep latency (component 2 PSQI)	1.0 (1.0–2.0)	1.0 (1.0–2.0)	0.821
	Sleep duration (component 3 PSQI)	1.0 (0.0–2.0)	2.0 (1.0–2.5)	0.200
	Sleep efficiency (component 4 PSQI)	1.0 (0.0–2.0)	1.0 (0.0–3.0)	0.594
	Sleep disturbance (component 5 PSQI)	1.0 (1.0–2.0)	2.0 (1.0–2.0)	0.029
	Use of sleep medication (component 6 PSQI) [yes (%)/no (%)]	24(80%)/6(20%)	22(73.3%)/8(27.7%)	0.542
	Daytime dysfunction (component 7 PSQI)	2.0 (1.0–3.0)	1.0 (1.0–2.5)	0.452
	Reported time spent lying in bed in PSQI (min)	420.0 (390.0–540.0)	450.0 (360.0–525.0)	0.521
	Subjective Sleep Efficiency in PSQI (%)	79.9 ± 15.3	76.6 ± 15.4	0.403
	BDI	14.0 (7.0–20.0)	11.0 (8.0–16.5)	0.885
Protein Concentration	BDNF evening [ng/mL]	13.4 (6.8–22.8)	9.0 (6.1–25.2)	0.442
	BDNF morning [ng/mL]	19.7 (7.7–24.7)	9.2 (6.6–23.4)	0.626
	proBDNF evening [ng/mL]	6.1 (3.5–10.1)	4.7 (2.5–11.8)	0.559
	proBDNF morning [ng/mL]	7.7 (3.2–11.2)	5.9 (3.0–11.1)	0.496

AHI—apnea-hypopnea index; AIS—Athens Insomnia Scale; BDI—Beck Depression Inventory; BDNF—brain-derived neurotrophic factor; BMI—body mass index; NREM—non rapid eye movement sleep; OSA—obstructive sleep apnea, proBDNF—premature brain-derived neurotrophic factor; PSQI—Pittsburgh Sleep Quality Index; REM—rapid eye movement sleep; SpO_2_—oxygen saturation; TST—total sleep time.

**Table 2:** Baseline characteristics of the study subgroups.

		OSA Group (*n* = 30)			OSA Group (*n* = 30)			OSA Group (*n* = 30)		
		AIS (−) Group (≤5; *n* = 6)	AIS (+) Group (>5; *n* = 24)	*p*-Value	PSQI (−) Group (≤5; *n* = 5)	PSQI (+) Group (>5; *n* = 25)	*p*-Value	BDI (−) Group (≤19; *n* = 25)	BDI (+) Group (>19; *n* = 5)	*p*-Value
Demographic data	Sex [M (%)/F(%)]	6 (100%)/0 (0.0%)	21 (87.5%)/3(12.5%)	0.361	5 (100%)/0(0.0%)	22 (88.0%)/3 (12.0%)	0.414	24 92.0%)/1(8.0%)	2(40.0%)/3(60.0%)	0.064
	Age [years]	57.7 ± 10.1	57.5± 10.2	0.979	56.4 ± 10.7	57.8 ± 10.1	0.780	56.7 ±10.0	61.8 ± 9.8	0.307
	BMI [kg/m2]	35.5 ± 4.5	35.6 ± 5.7	0.968	36.1 ± 4.8	35.5 ± 5.6	0.835	35.2 ± 5.6	37.5 ± 3.8	0.399
Polysomnography	TST (h)	6.1 ± 0.9	5.8 ± 1.2	0.575	6.4 ± 0.8	5.8 ± 1.2	0.284	5.9 ± 1.1	5.7 ± 1.1	0.701
	REM (h)	1.2 ± 0.7	1.2 ± 0.6	0.763	1.4 ± 0.7	1.1 ± 0.6	0.399	2.8 ± 1.6	1.5 ± 1.0	0.205
	NREM (h)	4.9 ± 0.6	4.7 ±0.9	0.613	5.0 ± 0.6	4.7 ± 0.9	0.439	4.8 ± 0.9	4.2 ± 0.7	0.170
	Sleep Efficiency (%)	80.3 ± 15.3	79.2 ± 14.2	0.894	87.2 ± 8.1	11.8 ± 11.7	0.305	80.0 ± 15.1	77.9 ± 11.3	0.808
	Sleep onset latency (min)	13.0 (5.5–27.4)	16.8 (8.1–30.1)	0.679	11.1 ± 5.9	23.9 ± 24.5	0.029	15.5 (7.3–25.3)	30.5 (9.8–78.5)	0.033
	Sleep Maintenance Efficiency (%)	88.7 (74.1–94.9)	89.1 (76.7–92.2)	0.808	91.1 ± 5.1	23.9 ± 24.7	0.115	89.1 (77.2–93.7)	88.0 (73.1–92.4)	0.992
	REM latency (min)	86.8 ± 33.0	120.2 ± 70.22	0.107	81.5 ± 33.8	68.7 ± 119.9	0.085	117.0 ± 69.1	96.0 ± 44.1	0.521
	arousal index (events/h)	25.9 (23.0–28.2)	23.6 (16.6–36.5)	0.710	24.7 ± 1.9	29.2 ± 18.2	0.235	29.2 ± 17.3	24.6 ± 13.9	0.573
	AHI	48.3 (37.1–64.0)	49.7 (37.7–73.2)	0.609	46.3 (36.6–63.3)	51.5 (38.1–72.2)	0.519	53.0 ± 19.6	67.0 ± 34.9	0.213
	AHI in REM sleep (events/h)	42.7 (30.8–65.8)	47.4 (27.2–65.4)	0.636	39.5 (22.5–56.2)	50.9 (27.2–65.9)	0.407	45.2 ± 22.9	64.3 ± 36.5	0.134
	AHI in NREM sleep (events/h)	46.2 (33.0–64.2)	47.6 (37.9–70.6)	0.557	37.3 (32.7–67.4)	47.6 (38.0–69.8)	0.471	52.0 ± 20.5	67.8 ± 35.8	0.177
	Total number of desaturations	320.0 (249.5–498.5)	211.0 (188.5–383.5)	0.707	347.0 (235.0–377.0)	229.5 (194.8–381.3)	0.605	235.0 (188.5–363.0)	318.0 (215.8–727.0)	0.254
	Desaturation Index	53.3 ± 15.5	57.5 ± 28.5	0.730	51.7 ± 16.8	57.6 ± 27.9	0.653	54.8 ± 25.1	65.9 ± 33.1	0.398
	Mean SpO2 of desaturations during sleep	85.4 (84.4–86.9)	86.7 (81.2–88.8)	0.376	85.1 (83.9–87.8)	86.4 (81.3–88.7)	0.422	86.0 (82.5–88.2)	85.4 (81.6–93.5)	0.700
Questionaires	AIS	N/A	N/A	N/A	2.5 (0.5–3.8)	12.0 (8.0–14.0)	<0.001	10.0 (6.3–13.0)	14.0 (10.5–15.0)	0.046
	PSQI	5.0 (2.5–8.5)	11.0 (7.5–13.0)	0.001	N/A	N/A	N/A	10.5 (6.3–12.0)	13.0 (10.0–14.5)	0.090
	Sleep latency (component 2 PSQI)	0.0 (0.0–0.5)	2.0 (1.0–2.0)	<0.001	N/A	N/A	N/A	2.0 (1.0–2.0)	2.0 (1.0–2.5)	0.356
	Sleep duration (component 3 PSQI)	1.0 (0.5–1.0)	2.0 (1.0–2.0)	0.014	N/A	N/A	N/A	1.0 (1.0–2.0)	2.0 (0.5–2.5)	0.690
	Sleep efficiency (component 4 PSQI)	0.0 (0.0–0.5)	2.0 (1.0–3.0)	<0.001	N/A	N/A	N/A	2.0 (1.0–2.8)	2.0 (1.0–2.5)	0.792
	Sleep disturbance (component 5 PSQI)	0.0 (0.0–1.0)	1.5 (0.0–3.0)	0.044	N/A	N/A	N/A	1.0 (0.0–2.8)	2.0 (0.5–3.0)	0.385
	Use of sleep medication (component 6 PSQI) [yes (%)/no (%)]	6(100%)/0(0%)	18(75%)/6(25%)	0.171	N/A	N/A	N/A	20(83.3%)/4(16.7%)	4(66.7%)/2(33.3%)	0.361
	Daytime dysfunction (component 7 PSQI)	0.0 (0.0–2.5)	0.0 (0.0–0.8)	0.327	N/A	N/A	N/A	0.0 (0.0–0.0)	1.0 (0.0–2.0)	0.327
	Sleep latency (component 2 PSQI)	1.0 (0.5–3.0)	1.0 (1.0–2.0)	0.973	N/A	N/A	N/A	1.0 (1.0–2.0)	2.0 (1.5–3.0)	0.127
	Reported time spent lying in bed in PSQI (min)	534.0 ± 57.7	425.4 ± 99.2	0.027	N/A	N/A	N/A	77.4 ± 15.3	72.6 ± 17.1	0.726
	Subjective Sleep Efficiency in PSQI (%)	90.2 ± 13.8	73.7 ± 14.4	0.027	N/A	N/A	N/A	441.0 ± 105.2	459.0 ± 89.8	0.541
	BDI	7.0 (2.0–10.5)	14.0 (8.5–19.5)	0.013	9.0 (5.0–9.0)	13.5 (8.0–18.3)	0.033	N/A	N/A	N/A
Protein Concentration	BDNF evening [ng/mL]	6.6 (6.2–8.1)	18.8 (6.1–26.7)	<0.001 *	6.6 (6.4–8.1)	17.5 (6.0–26.4)	<0.001 *	9.8 (6.3–26.4)	6.1 (4.9–24.9)	0.303
	BDNF morning [ng/mL]	14.0 (8.0–17.8)	14.4 (6.6–28.0)	0.277	13.4 (6.9–17.3)	16.2 (6.6–26.7)	0.222	16.2 (8.0–26.7)	6.6 (5.2–14.3)	0.047 **
	proBDNF evening [ng/mL]	3.1 (2.1–3.8)	9.7 (2.5–28.0)	<0.001 *	2.9 (2.0–4.0)	8.6 (2.5–13.9)	<0.001 *	4.9 (2.6–13.9)	2.6 (2.0–10.8)	0.388
	proBDNF morning [ng/mL]	6.0 (3.6–9.2)	5.8 (2.7–12.3)	0.630	5.8 (2.7–8.6)	6.1 (2.9–12.0)	0.504	7.8 ± 5.1	3.5 ± 1.7	0.003 **

AHI—apnea-hypopnea index; AIS—Athens Insomnia Scale; BDI—Beck Depression Inventory; BDNF—brain-derived neurotrophic factor; BMI—body mass index; N/A – not applicable; NREM—non-rapid eye movement sleep; OSA—obstructive sleep apnea, proBDNF—premature brain-derived neurotrophic factor; PSQI—Pittsburgh Sleep Quality Index; REM—rapid eye movement sleep; SpO_2_—oxygen saturation; TST—total sleep time; * small effect size; ** medium effect size.

## Data Availability

Data will be made available upon request.

## Abbreviations

**Abbreviations**	**Full Name**
7:8-DHF	7,8-dihydroxyflavone
AHI	apnea-hypopnea index
AIS	Athens Insomnia Scale
Akt	AKT serine/threonine kinase
BDI	Beck Depression Inventory
BDNF	brain-derived neurotrophic factor
BMAL1	aryl hydrocarbon receptor nuclear translocator-like protein 1
CIH	chronic intermittent hypoxia
CPAP	continous positive airway pressure
CREB	CREB binding protein
EDS	excessive daytime sleepiness
HIF-1	hypoxia-inducable factor 1
HPA	hypothalamus-pituitary-adrenal glands axis
IH	intermittent hypoxia
IL1	interleukin 1
IQR	interquartile range
JNK	mitogen-activated protein kinase 8
LTP	long-term potentiation
MAPK	mitogen-activated protein kinase 1
MDD	major depressive disorder
NREM	non-rapid eye movement
NT	neurotrophin
OSA	obstructive sleep apnea
PER1	period 1 protein
PI3K	-4,5-bisphosphate 3-kinase catalytic subunit
PKC	protein kinase C
PLC	phospholipase C
proBDNF	premature BDNF
PSG	polysomnography
PSQI	Pittsburgh Sleep Quality Index
Ras	KRAS proto-oncogene
REM	rapid eye movement
ROS	reactive oxygen species
SAPK	mitogen-activated protein kinase 4
SpO_2_	hemoglobin oxygen saturation
TrkB	neurotrophic receptor tyrosine kinase 2

## References

[1] Gottlieb D.J., Punjabi N.M. (2020). Diagnosis and Management of Obstructive Sleep Apnea: A Review. JAMA.

[2] Hosseini H., Homayouni-Tabrizi M., Amiri H., Safari-Faramani R., Moradi M.-T., Fadaei R., Khazaie H. (2022). The effect of continuous positive airway pressure on total antioxidant capacity in obstructive sleep apnea: A systematic review and meta-analysis. Sleep Breath..

[3] Gabryelska A., Chrzanowski J., Sochal M., Kaczmarski P., Turkiewicz S., Ditmer M., Karuga F.F., Czupryniak L., Białasiewicz P. (2021). Nocturnal Oxygen Saturation Parameters as Independent Risk Factors for Type 2 Diabetes Mellitus among Obstructive Sleep Apnea Patients. J. Clin. Med..

[4] Heinzer R., Vat S., Marques-Vidal P., Marti-Soler H., Andries D., Tobback N., Mooser V., Preisig M., Malhotra A., Waeber G. (2015). Prevalence of sleep-disordered breathing in the general population: THE HypnoLaus study. Lancet Respir. Med..

[5] Tietjens J.R., Claman D., Kezirian E.J., De Marco T., Mirzayan A., Sadroonri B., Goldberg A.N., Long C., Gerstenfeld E.P., Yeghiazarians Y. (2019). Obstructive Sleep Apnea in Cardiovascular Disease: A Review of the Literature and Proposed Multidisciplinary Clinical Management Strategy. J. Am. Heart Assoc..

[6] Yeghiazarians Y., Jneid H., Tietjens J.R., Redline S., Brown D.L., El-Sherif N., Mehra R., Bozkurt B., Ndumele C.E., Somers V.K. (2021). Obstructive Sleep Apnea and Cardiovascular Disease: A Scientific Statement From the American Heart Association. Circulation.

[7] Reutrakul S., Mokhlesi B. (2017). Obstructive Sleep Apnea and Diabetes: A State of the Art Review. Chest.

[8] Mok Y., Tan C.W., Wong H.S., How C.H., Tan K.L.A., Hsu P.P. (2017). Obstructive sleep apnoea and Type 2 diabetes mellitus: Are they connected?. Singap. Med. J..

[9] Ditmer M., Gabryelska A., Turkiewicz S., Białasiewicz P., Małecka-Wojciesko E., Sochal M. (2021). Sleep Problems in Chronic Inflammatory Diseases: Prevalence, Treatment, and New Perspectives: A Narrative Review. J. Clin. Med..

[10] Gabryelska A., Sochal M., Wasik B., Białasiewicz P. (2018). Patients With Obstructive Sleep Apnea Are Over Four Times More Likely to Suffer From Psoriasis Than the General Population. J. Clin. Sleep Med..

[11] Kuna K., Szewczyk K., Gabryelska A., Białasiewicz P., Ditmer M., Strzelecki D., Sochal M. (2022). Potential Role of Sleep Deficiency in Inducing Immune Dysfunction. Biomedicines.

[12] Kang J.-H., Lin H.-C. (2012). Obstructive sleep apnea and the risk of autoimmune diseases: A longitudinal population-based study. Sleep Med..

[13] Parish J.M. (2013). Genetic and immunologic aspects of sleep and sleep disorders. Chest.

[14] Verbraecken J. (2022). More than sleepiness: Prevalence and relevance of nonclassical symptoms of obstructive sleep apnea. Curr. Opin. Pulm. Med..

[15] Gabryelska A., Sochal M., Wasik B., Szczepanowski P., Białasiewicz P. (2021). Factors Affecting Long-Term Compliance of CPAP Treatment—A Single Centre Experience. J. Clin. Med..

[16] Luyster F.S., Buysse D.J., Strollo P.J.J. (2010). Comorbid insomnia and obstructive sleep apnea: Challenges for clinical practice and research. J. Clin. sleep Med..

[17] Cistulli P.A., Armitstead J., Pepin J.-L., Woehrle H., Nunez C.M., Benjafield A., Malhotra A. (2019). Short-term CPAP adherence in obstructive sleep apnea: A big data analysis using real world data. Sleep Med..

[18] Lechat B., Appleton S., Melaku Y.A., Hansen K., McEvoy R.D., Adams R., Catcheside P., Lack L., Eckert D.J., Sweetman A. (2022). Comorbid insomnia and sleep apnoea is associated with all-cause mortality. Eur. Respir. J..

[19] Jehan S., Auguste E., Pandi-Perumal S.R., Kalinowski J., Myers A.K., Zizi F., Rajanna M.G., Jean-Louis G., McFarlane S.I. (2017). Depression, Obstructive Sleep Apnea and Psychosocial Health. Sleep Med. Disord. Int. J..

[20] Reddy A., Mansuri Z., Vadukapuram R., Trivedi C. (2022). Increased Suicidality and Worse Outcomes in MDD Patients With OSA: A Nationwide Inpatient Analysis of 11 Years From 2006 to 2017. J. Acad. Consult. Psychiatry.

[21] Sochal M., Ditmer M., Gabryelska A., Białasiewicz P. (2022). The Role of Brain-Derived Neurotrophic Factor in Immune-Related Diseases: A Narrative Review. J. Clin. Med..

[22] Shi X.-J., Du Y., Lei-Chen, Li X.-S., Yao C.-Q., Cheng Y. (2022). Effects of brain-derived neurotrophic factor (BDNF) on the Schizophrenia model of animals. J. Psychiatr. Res..

[23] Meshkat S., Alnefeesi Y., Jawad M.Y., Di Vincenzo J.D., Rodrigues N.B., Ceban F., Lui L.M., McIntyre R.S., Rosenblat J.D. (2022). Brain-Derived Neurotrophic Factor (BDNF) as a biomarker of treatment response in patients with Treatment Resistant Depression (TRD): A systematic review & meta-analysis. Psychiatry Res..

[24] Miuli A., Mancusi G., Pettorruso M., Di Carlo F., Clemente K., Di Meo I., D’Andrea A., Pernaci G., Di Crosta T., d’Andrea G. (2022). Impact of sleep disorders and disease duration on neurotrophins levels in cocaine use disorder. Neurosci. Lett..

[25] Nordvall G., Forsell P., Sandin J. (2022). Neurotrophin-targeted therapeutics: A gateway to cognition and more?. Drug Discov. Today.

[26] Xie H., Yung W. (2012). Chronic intermittent hypoxia-induced deficits in synaptic plasticity and neurocognitive functions: A role for brain-derived neurotrophic factor. Acta Pharmacol. Sin..

[27] Watson A.J., Henson K., Dorsey S.G., Frank M.G. (2015). The truncated TrkB receptor influences mammalian sleep. Am. J. Physiol. Regul. Integr. Comp. Physiol..

[28] Castrén E., Monteggia L.M. (2021). Brain-Derived Neurotrophic Factor Signaling in Depression and Antidepressant Action. Biol. Psychiatry.

[29] Sochal M., Małecka-Panas E., Gabryelska A., Fichna J., Talar-Wojnarowska R., Szmyd B., Białasiewicz P. (2021). Brain-derived neurotrophic factor is elevated in the blood serum of Crohn’s disease patients, but is not influenced by anti-TNF-α treatment—A pilot study. Neurogastroenterol. Motil. Off. J. Eur. Gastrointest. Motil. Soc..

[30] Panaree B., Chantana M., Wasana S., Chairat N. (2011). Effects of obstructive sleep apnea on serum brain-derived neurotrophic factor protein, cortisol, and lipid levels. Sleep Breath..

[31] Campos-Rodriguez F., Asensio-Cruz M.I., Cordero-Guevara J., Jurado-Gamez B., Carmona-Bernal C., Gonzalez-Martinez M., Troncoso M.F., Sanchez-Lopez V., Arellano-Orden E., Garcia-Sanchez M.I. (2019). Effect of continuous positive airway pressure on inflammatory, antioxidant, and depression biomarkers in women with obstructive sleep apnea: A randomized controlled trial. Sleep.

[32] Kaminska M., O’Sullivan M., Mery V.P., Lafontaine A.L., Robinson A., Gros P., Martin J.G., Benedetti A., Kimoff R.J. (2022). Inflammatory markers and BDNF in obstructive sleep apnea (OSA) in Parkinson’s disease (PD). Sleep Med..

[33] Flores K.R., Viccaro F., Aquilini M., Scarpino S., Ronchetti F., Mancini R., Di Napoli A., Scozzi D., Ricci A. (2020). Protective role of brain derived neurotrophic factor (BDNF) in obstructive sleep apnea syndrome (OSAS) patients. PLoS ONE.

[34] Kapur V.K., Auckley D.H., Chowdhuri S., Kuhlmann D.C., Mehra R., Ramar K., Harrod C.G. (2017). Clinical practice guideline for diagnostic testing for adult obstructive sleep apnea: An American academy of sleep medicine clinical practice guideline. J. Clin. Sleep Med..

[35] Smyka M., Kosińska-Kaczyńska K., Sochacki-Wójcicka N., Zgliczyńska M., Wielgoś M. (2021). Sleep quality according to the Pittsburgh Sleep Quality Index in over 7000 pregnant women in Poland. Sleep Biol. Rhythm..

[36] Backhaus J., Junghanns K., Broocks A., Riemann D., Hohagen F. (2002). Test-retest reliability and validity of the Pittsburgh Sleep Quality Index in primary insomnia. J. Psychosom. Res..

[37] Chabowski M., Łuczak J., Dudek K., Jankowska-Polańska B. (2019). Sleep disorders and adherence to inhalation therapy in patients with chronic obstructive pulmonary disease. Adv. Exp. Med. Biol..

[38] Mollayeva T., Thurairajah P., Burton K., Mollayeva S., Shapiro C.M., Colantonio A. (2016). The Pittsburgh sleep quality index as a screening tool for sleep dysfunction in clinical and non-clinical samples: A systematic review and meta-analysis. Sleep Med. Rev..

[39] Staniszewska A., Mąka A., Religioni U., Olejniczak D. (2017). Sleep disturbances among patients with epilepsy. Neuropsychiatr. Dis. Treat..

[40] Smarr K.L., Keefer A.L. (2011). Measures of depression and depressive symptoms: Beck Depression Inventory-II (BDI-II), Center for Epidemiologic Studies Depression Scale (CES-D), Geriatric Depression Scale (GDS), Hospital Anxiety and Depression Scale (HADS), and Patient Health Questionn. Arthritis Care Res..

[41] Mirghafourvand M., Charandabi S.M.A., lak T.B., Aliasghari F. (2018). Predictors of Depression in Iranian Women with Polycystic Ovarian Syndrome. Community Ment. Health J..

[42] Beck A.T., Steer R.A., Brown G. (1996). Beck depression inventory–II. Psychol. Assess..

[43] Dabson K.S., MOHAMMAD K.P. (2007). Psychometric characteristics of Beck depression inventory–II in patients with major depressive disorder. Brain Behav..

[44] Wiglusz M.S., Landowski J., Michalak L., Cubała W.J. (2017). Validation of the Polish version of the Beck Depression Inventory in patients with epilepsy. Epilepsy Behav..

[45] Kokoszka A., Cichoń E., Obrębski M., Kiejna A., Rajba B. (2020). Cut-off points for Polish-language versions of depression screening tools among patients with Type 2 diabetes. Prim. Care Diabetes.

[46] Soldatos C.R., Dikeos D.G., Paparrigopoulos T.J. (2000). Athens Insomnia Scale: Validation of an instrument based on ICD-10 criteria. J. Psychosom. Res..

[47] Fornal-Pawłowska M., Wołyńczyk-Gmaj D., Szelenberger W. (2011). Validation of the Polish version of the Athens Insomnia Scale. Psychiatr. Pol..

[48] Fang Y.-Y., Luo M., Yue S., Han Y., Zhang H.-J., Zhou Y.-H., Liu K., Liu H.-G. (2022). 7,8-Dihydroxyflavone protects retinal ganglion cells against chronic intermittent hypoxia-induced oxidative stress damage via activation of the BDNF/TrkB signaling pathway. Sleep Breath..

[49] An J.R., Zhao Y.S., Luo L.F., Guan P., Tan M., Ji E.S. (2020). Huperzine A, reduces brain iron overload and alleviates cognitive deficit in mice exposed to chronic intermittent hypoxia. Life Sci..

[50] Yang L., Zhao J., Qu Y., Sun Q., Li T.-T., Yan M.-L., Duan M.-J., Li K.-X., Wang Y.-R., Huang S.-Y. (2020). Metoprolol prevents neuronal dendrite remodeling in a canine model of chronic obstructive sleep apnea. Acta Pharmacol. Sin..

[51] Wu X., Lu H., Hu L., Gong W., Wang J., Fu C., Liu Z., Li S. (2017). Chronic intermittent hypoxia affects endogenous serotonergic inputs and expression of synaptic proteins in rat hypoglossal nucleus. Am. J. Transl. Res..

[52] Zhang J., Guo X., Shi Y., Ma J., Wang G. (2014). Intermittent hypoxia with or without hypercapnia is associated with tumorigenesis by decreasing the expression of brain derived neurotrophic factor and miR-34a in rats. Chin. Med. J..

[53] Xie H., Leung K.-L., Chen L., Chan Y.-S., Ng P.-C., Fok T.-F., Wing Y.-K., Ke Y., Li A.M., Yung W.-H. (2010). Brain-derived neurotrophic factor rescues and prevents chronic intermittent hypoxia-induced impairment of hippocampal long-term synaptic plasticity. Neurobiol. Dis..

[54] Zhang C.Q., Yi S., Chen B.B., Cui P.P., Wang Y., Li Y.Z. (2021). mTOR/NF-κB signaling pathway protects hippocampal neurons from injury induced by intermittent hypoxia in rats. Int. J. Neurosci..

[55] Turkiewicz S., Ditmer M., Sochal M., Białasiewicz P., Strzelecki D., Gabryelska A. (2021). Obstructive Sleep Apnea as an Acceleration Trigger of Cellular Senescence Processes through Telomere Shortening. Int. J. Mol. Sci..

[56] Tang S., Machaalani R., Waters K.A. (2008). Brain-derived neurotrophic factor (BDNF) and TrkB in the piglet brainstem after post-natal nicotine and intermittent hypercapnic hypoxia. Brain Res..

[57] Makhout S., Vermeiren E., Van De Maele K., Bruyndonckx L., De Winter B.Y., Van Hoorenbeeck K., Verhulst S.L., Van Eyck A. (2022). The Role of Brain-Derived Neurotrophic Factor in Obstructive Sleep Apnea and Endothelial Function in a Pediatric Population With Obesity. Front. Med..

[58] Campos-Rodriguez F., Cordero-Guevara J., Asensio-Cruz M.I., Sanchez-Armengol A., Sanchez-Lopez V., Arellano-Orden E., Gozal D., Martinez-Garcia M.A. (2021). Interleukin 6 as a marker of depression in women with sleep apnea. J. Sleep Res..

[59] Gabryelska A., Sochal M. (2022). Evaluation of HIF-1 Involvement in the BDNF and ProBDNF Signaling Pathways among Obstructive Sleep Apnea Patients. Int. J. Mol. Sci..

[60] Shah F., Forsgren S., Holmlund T., Levring Jäghagen E., Berggren D., Franklin K.A., Stål P. (2019). Neurotrophic factor BDNF is upregulated in soft palate muscles of snorers and sleep apnea patients. Laryngoscope Investig. Otolaryngol..

[61] Svanborg E. (2001). Upper airway nerve lesions in obstructive sleep apnea. Am. J. Respir. Crit. Care Med..

[62] Shah F., Holmlund T., Jäghagen E.L., Berggren D., Franklin K., Forsgren S., Stål P. (2018). Axon and Schwann Cell Degeneration in Nerves of Upper Airway Relates to Pharyngeal Dysfunction in Snorers and Patients With Sleep Apnea. Chest.

[63] Arslan B., Şemsi R., İriz A., Dinçel A.S. (2021). The evaluation of serum brain-derived neurotrophic factor and neurofilament light chain levels in patients with obstructive sleep apnea syndrome. Laryngoscope Investig. Otolaryngol..

[64] Targa A., Dakterzada F., Benítez I., López R., Pujol M., Dalmases M., Arias A., Sánchez-de-la-Torre M., Zetterberg H., Blennow K. (2021). Decrease in sleep depth is associated with higher cerebrospinal fluid neurofilament light levels in patients with Alzheimer’s disease. Sleep.

[65] Gabryelska A., Szmyd B., Panek M., Szemraj J., Kuna P., Białasiewicz P. (2020). Serum hypoxia-inducible factor-1α protein level as a diagnostic marker of obstructive sleep apnea. Pol. Arch. Intern. Med..

[66] Gabryelska A., Szmyd B., Szemraj J., Stawski R., Sochal M., Białasiewicz P. (2020). Patients with obstructive sleep apnea present with chronic upregulation of serum HIF-1α protein. J. Clin. sleep Med..

[67] Gabryelska A., Stawski R., Sochal M. (2020). Influence of one-night CPAP therapy on the changes of HIF-1α protein in OSA patients—A pilot study. J. Sleep Res..

[68] Lu D., Li N., Yao X., Zhou L. (2017). Potential inflammatory markers in obstructive sleep apnea-hypopnea syndrome. Bosn. J. basic Med. Sci..

[69] Qaid E.Y.A., Zulkipli N.N., Zakaria R., Ahmad A.H., Othman Z., Muthuraju S., Sasongko T.H. (2021). The role of mTOR signalling pathway in hypoxia-induced cognitive impairment. Int. J. Neurosci..

[70] More C.E., Papp C., Harsanyi S., Gesztelyi R., Mikaczo A., Tajti G., Kardos L., Seres I., Lorincz H., Csapo K. (2019). Altered irisin/BDNF axis parallels excessive daytime sleepiness in obstructive sleep apnea patients. Respir. Res..

[71] Fan T.-T., Chen W.-H., Shi L., Lin X., Tabarak S., Chen S.-J., Que J.-Y., Bao Y.-P., Tang X.-D., Shi J. (2019). Objective sleep duration is associated with cognitive deficits in primary insomnia: BDNF may play a role. Sleep.

[72] Mikoteit T., Brand S., Eckert A., Holsboer-Trachsler E., Beck J. (2019). Brain-derived neurotrophic factor is a biomarker for subjective insomnia but not objectively assessable poor sleep continuity. J. Psychiatr. Res..

[73] Giese M., Unternährer E., Hüttig H., Beck J., Brand S., Calabrese P., Holsboer-Trachsler E., Eckert A. (2014). BDNF: An indicator of insomnia?. Mol. Psychiatry.

[74] Backhaus J., Junghanns K., Hohagen F. (2004). Sleep disturbances are correlated with decreased morning awakening salivary cortisol. Psychoneuroendocrinology.

[75] Riemann D., Klein T., Rodenbeck A., Feige B., Horny A., Hummel R., Weske G., Al-Shajlawi A., Voderholzer U. (2002). Nocturnal cortisol and melatonin secretion in primary insomnia. Psychiatry Res..

[76] Claro A.E., Palanza C., Mazza M., Rizzi A., Tartaglione L., Marano G., Muti-Schuenemann G., Rigoni M., Muti P., Pontecorvi A. (2022). Why do we not reverse the path? Stress can cause depression, reduction of brain-derived neurotrophic factor and increased inflammation. World J. Psychiatry.

[77] Gabryelska A., Turkiewicz S., Karuga F.F., Sochal M., Strzelecki D., Białasiewicz P. (2022). Disruption of Circadian Rhythm Genes in Obstructive Sleep Apnea Patients—Possible Mechanisms Involved and Clinical Implication. Int. J. Mol. Sci..

[78] Gabryelska A., Sochal M., Turkiewicz S., Białasiewicz P. (2020). Relationship between HIF-1 and Circadian Clock Proteins in Obstructive Sleep Apnea Patients—Preliminary Study. J. Clin. Med..

[79] Cain S.W., Chang A.-M., Vlasac I., Tare A., Anderson C., Czeisler C.A., Saxena R. (2017). Circadian Rhythms in Plasma Brain-derived Neurotrophic Factor Differ in Men and Women. J. Biol. Rhythm..

[80] Wall A.M., Corcoran A.E., O’Halloran K.D., O’Connor J.J. (2014). Effects of prolyl-hydroxylase inhibition and chronic intermittent hypoxia on synaptic transmission and plasticity in the rat CA1 and dentate gyrus. Neurobiol. Dis..

